# Splenic Infarction in a Postpartum Patient with COVID-19

**DOI:** 10.1055/s-0041-1723783

**Published:** 2021-02-16

**Authors:** Syed Nazeer Mahmood, Yaser T. Dawod, Chee Man Chan

**Affiliations:** 1 Section of Pulmonary/Critical Care, Department of Medicine, MedStar Washington Hospital Center, Washington, District of Columbia, United States

Thrombotic events have been reported in patients with coronavirus disease 2019 (COVID-19). Pregnant and postpartum patients are also known to be hypercoagulable and those with COVID-19 may therefore be at an even higher risk of thrombotic complications. Here, we discuss a postpartum patient with COVID-19 who developed an isolated splenic infarction. While describing a rare thrombotic event, this case serves to alert physicians to the higher risk of thrombosis in pregnant or postpartum patients with COVID-19.

**Fig. 1:**
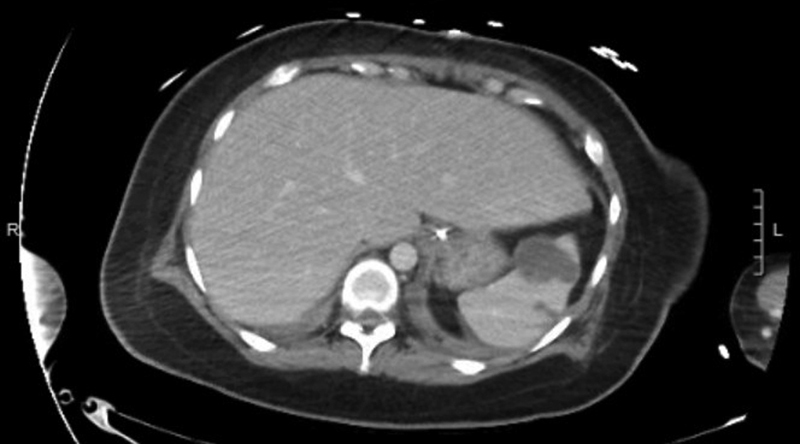
CT abdomen (axial view) with multiple infarcts in the spleen: largest lesion is 4.1 cm partially exophytic suspicious for hemorrhage into prior infarct. CT, computed tomography.

**Fig. 2:**
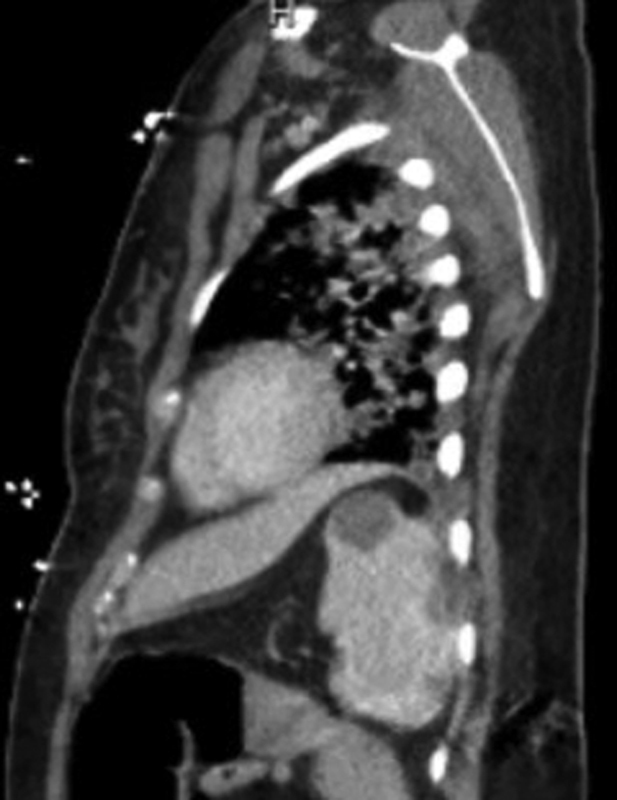
CT abdomen (sagittal view) with multiple infarcts in the spleen. CT, computed tomography.

A previously healthy 27-year-old female who was 37 weeks pregnant, with no past medical history presented to the hospital with complaints of fever, cough, and shortness of breath. She tested positive for COVID-19 and as she was improving, was discharged home. She presented to the hospital again 2 days later with worsening fevers and tachypnea and was transferred to our hospital for further management the same day. On day 2 of admission, she vaginally delivered a healthy baby girl. She continued to have worsening tachypnea and increasing oxygen requirements postpartum, eventually requiring endotracheal intubation on day 8. She was proned on the following day for refractory hypoxia with improvement in respiratory parameters. One dose of convalescent plasma (200 mL) was given on day 4 of admission and an 800 mg dose of tocilizumab on day 9 after concomitant bacterial infection was ruled out. She was also started on prophylactic heparin 5,000 units subcutaneously three times a day 2 days after delivery and continued throughout her stay. While intubated, the patient was hemodynamically stable and required only minimal vasopressor medications probably due to sedation effect. The patient's respiratory status started improving and ventilator weaning protocols were initiated. She tolerated supination on day 14. The patient had persistent low-grade fevers post-delivery with an elevation in her white blood cell in the 12 to 20 k/µl range. Multiple blood cultures were sent that were all negative. Her markers of inflammation were elevated initially after intubation with a ferritin of 486.9 ng/mL, C-reactive protein of 44.5 mg/L, and sedimentation rate of 75 mm/h. These markers improved progressively throughout her course. She did not have any laboratory abnormalities suggesting a hypercoagulable state with an initial fibrinogen of 368 mg/dL that stayed in the normal range throughout her stay. Prothrombin time, activated prothrombin time, and platelet count were also normal on admission and during her hospitalization. Her kidney function was also normal throughout in the 0.36 to 1.07 mg/dL range. Her D-dimer was elevated to >20 µg/mL immediately post-delivery but then decreased to 8.2 µg/mL a few days later. Her D-dimer level then gradually increased again, peaking at >20 µg/mL. On day 21, she complained of abdominal pain. In the setting of persistent fever and leukocytosis, there was concern for an abscess or intraabdominal catastrophe related to her delivery, such as, but not limited to, colonic pseudo-obstruction, infection, uterine involution, or foreign body retention. A computed tomography (CT) scan of the abdomen was therefore obtained that revealed multiple new hypodense splenic infarcts with possible prior hemorrhage into one infarct ( Figs. 1 , 2 ). There were no thromboembolic or infarctions noted on a prior CT chest and abdomen done earlier in the admission. Echocardiogram did not reveal any cardiac thrombi or vegetations that along with the negative blood cultures argued against embolic phenomena. The patient was started on therapeutic dose low molecular weight heparin (LMWH). She was successfully extubated on day 23 and was transitioned to apixaban prior to discharge. She is currently doing well.

Patients with COVID-19 are predisposed to thrombotic complications with cases of pulmonary embolism, deep vein thrombosis, and ischemic strokes reported. 1 The incidence of thrombotic complications is ∼31% in critically ill patients with COVID-19. 2 These complications are thought to be due to the presence of a hypercoagulable state from vascular damage in the setting of sepsis, from the virus itself, and from prolonged immobilization. 3 Abdominal visceral infarctions have been reported in COVID-19. We found only two case series with four patients in total who had splenic infarctions. Most of the cases had associated thrombosis of the intestines, kidney, or pulmonary vasculature and only two cases of isolated splenic infarction were reported. 4 5 6 Pregnancy is a known prothrombotic state as there is an elevation of procoagulant factors like factors II, VII, VIII, XII, and a reduction in anticoagulant factors such as protein S. There is also reduction in fibrinolysis due to increased activity of fibrinolytic inhibitors. These changes tend to persist up to 8 weeks after delivery. 7 Splenic infarcts are not a common complication in pregnancy unless the patient has associated conditions like infective endocarditis. 8 Other potential causes of splenic infarction include malignancy, vasculitis, and red blood cell disorders like sickle cell disease. 9 Our patient was relatively healthy and did not have a history of obesity, malignancy, autoimmune disorders or vasculitis, atrial fibrillation, and no red blood cell abnormalities were noted on peripheral smear. There was also no personal or family history of hypercoagulability. Patients with splenic infarctions have a varied presentation with fever and left upper quadrant abdominal pain being the most common. Patients may also have left sided chest pain, pleural effusion, hiccups due to diaphragmatic irritation, and abdominal distention. Ultrasound or CT of the abdomen can help with the diagnosis; however, a CT scan is preferred as it provides more information on adjoining organs and blood vessels. The management for splenic infarction involves treatment of the underlying disease state along with aggressive pain control. 9 When due to a hypercoagulable state, anticoagulation is the mainstay of treatment as was done with our patient. In pregnancy and in the postpartum period, LMWH is preferred and the use of apixaban is off label for splenic infarctions. In our patient, however, we transitioned to apixaban at discharge for patient ease. Further, given her recent pregnancy and COVID-19 infection, we believe that continued anticoagulation was necessary due to an acquired hypercoagulable state. Surgery can be considered in patients with splenic hemorrhage, aneurysm, or abscess but surgery results in a high risk of post splenectomy infections. 10 Our case is unique in that it describes a patient in her postpartum period with isolated splenic infarctions. Thrombotic complications of COVID-19 are known, but the impact on pregnant and postpartum patients remains unclear. While there are reports of pulmonary embolisms and arterial thrombi in this subgroup, the true incidence is still unknown. 11 Cases like ours suggest that pregnant patients with COVID-19 might be at a higher risk than nonpregnant COVID-19 patients for thrombotic events. With the high rate of thrombotic complications 2 and infrequent use of abdominal CT scans, the incidence of splenic and abdominal visceral infarctions is likely underestimated. Guidelines therefore recommend that unless there is an absolute contraindication, all COVID-19 patients should receive chemical venous thromboembolism prophylaxis. 12 These patients should also be assessed frequently for possible thrombotic events and the need for therapeutic anticoagulation. In pregnant or postpartum patients, there should be a lower threshold to evaluate for thrombotic complications and full-dose anticoagulation.
